# Mortality of alzheimer’s disease in Italy from 1980 to 2015

**DOI:** 10.1007/s10072-024-07791-3

**Published:** 2024-10-15

**Authors:** Daiana Bezzini, Carmelo L. Smeralda, Patrizio Pasqualetti, Stefano F. Cappa, Lucia Kundisova, Simone Rossi, Monica Ulivelli

**Affiliations:** 1 Department of Life Sciences, University of Siena, via Aldo Moro 2, Siena, 53100 SI Italy; 2 Department of Medicine, Surgery and Neuroscience, Unit of Neurology and Clinical Neurophysiology, Policlinico Le Scotte, University of Siena, Siena, Italy; 3 Health Statistics, University La Sapienza, Roma, Italy; 4 Institute for Advanced Study, IUSS, Pavia, Italy; 5 IRCCS Mondino Foundation, Pavia, Italy; 6 Postgradual School of Public Health, University of Siena, Siena, Italy

**Keywords:** Alzheimers’s disease, Epidemiology, Mortality, Degenerative diseases

## Abstract

**Objective:**

To evaluate mortality for Alzheimer’s Disease (AD) in Italy over more than three decades (1980–2015) and discuss the possible role of general and specific contributing factors.

**Methods:**

Mortality data were extracted by the Italian National Institute of Statistics: crude mortality rates were computed for sex and age, considering the whole country and its five main geographical sub-areas. Rates were standardized in two ways: directly (annual mortality rates AMRs) and indirectly (standardized mortality rates, SMRs). SMRs were then used to evaluate geographical differences; to study mortality trend, AMRs and joinpoint linear regression analysis were used.

**Results:**

Considering the entire period and the whole country, mortality rates were similar for females and males and for geographical regions, with the exception of the older age groups where mortality for AD in females slightly prevailed. In these older patients, a steep increase of mortality was seen starting from the current century. The increase in male mortality mirrored the national trend in North-West and Central Italy, but not in North-East, South, and the Islands, where it did not surge until the mid to late 1990s.

**Conclusions:**

the general increase of mortality is in line with international data and it reflects the increasing prevalence of the disease, likely due to increasing longevity, and to improvements in diagnostic accuracy. In addition, the accuracy of death certificate compilation could account for both geographical and temporal differences. Currently available drugs for AD do not seem to have an impact on mortality rates.

## Introduction

With more than 55 million people affected and nearly 10 million new cases per year, dementia is one of the most widespread neurological conditions and the seventh cause of death worldwide [1, 2]. In Europe, it is estimated that almost 9 millions of people were affected by dementia in 2019 (1.73% of the European population), of which about 1.300.000 in Italy (2.12% of the Italian population) [3].

The most common form of dementia is Alzheimer’s disease (AD), which contributes to 60–70% of all dementia cases [4].

In Europe, around 7 million people were estimated to be affected by AD in 2019 [3] with higher prevalence and incidence rates in women and in older subjects [5]. In Italy, about 150,000 new AD cases per year are expected [6].

Concerning mortality, in 2019 the Global Burden of Disease study estimated 1.62 million (0.41–4.21) deaths due to dementia, most of them among women (1.06 million [0.27–2.71] vs. 0.56 million in men [0.14–1.51]) [2]. In addition, the same study highlighted a large increase in all-age mortality rates (100.1% [89.1–117.5]), due to population aging, between 1990 and 2019 [2]. In Italy, AD represents the first cause of death among neurodegenerative disorders and the sixth cause among all diseases but, in contrast to all other conditions included in the ranking, it showed a constant increasing trend in the period 2003–2014 that can be largely explained by the aging of the population, with the consequent increase of age-associated degenerative diseases [7].

Italian national mortality data are routinely recorded in the Italian National Institute of Statistics (ISTAT) database. Through its consultation, mortality rates for specific diseases can be obtained, allowing researchers and clinicians to estimate epidemiological trends at both national and regional levels.

The aim of the present study is to assess mortality due to AD in Italy over more than three decades (i.e., from 1980 to 2015) and its distribution in the five main geographical areas of the country. Such 35 year time-span has been considered in order to compare AD data with Parkinson’s disease in the same period [8].

## Materials and methods

Mortality data, for the analysed period, were taken from the ISTAT database in the dis-aggregated form, in observance of the privacy law. As this is an observational descriptive study, based on anonymized data from ISTAT, neither an Institutional Review Board approval nor informed consent are required. The initial causes of death, ascertained by physicians belonging to the National Health System, were coded using the 9th revision of the International Classification of Diseases (ICD-9) until 2003 and ICD-10 from 2004 on. Starting from 1980, year of switch from ICD-8 to 9, we included all available death certificates mentioning AD as the underlying cause of death (ICD-9 code: 294.1 and 331.0; ICD-10 code: G30.0, G30.1, G30.8, G30.9).

Italy, formed by 20 regions, can be divided in five main geographical areas: North-West (NW), North-East (NE), Centre (C), South (S) and Islands (Sicily and Sardinia) (I). Firstly, we calculated both overall and annual crude mortality rates (CR) (number of deaths divided by resident population), for the whole country and then for each geographical area. In addition, we calculated the annual mortality rate by sex and age class. In order to identify, in the entire period, geographical areas with higher/lower mortality in comparison with the national rate, the standardized mortality ratios (SMRs) were computed (indirect standardization). SMRs were computed separately for each sex and were calculated as the ratio between the observed and expected number of deaths, using as expected deaths those observed for the whole country. The areas with SMR > 100 were those with higher mortality, while SMR < 100 reported a lower mortality than the national rate. The statistical significance was set on 95% confidence intervals.

Secondly, the mortality trend during the study period (1980–2015) was computed for the five geographical areas through direct standardization method: the annual mortality rates standardized by age and sex (AMRs) were calculated, using as standard population the European one [Eurostat].

Finally, the joinpoint regression model (Joinpoint software Version 4.5.0.1 - Statistical Research and Applications Branch, National Cancer Institute, available for free online) was applied to estimate the annual percent change in mortality (APC, expressed as %) and to identify the moments in time when the significant change in mortality in terms of increase or decrease occurred (the “joinpoint”). In order to establish the statistical significance of the results, 5% level was set.

## Results

**Fig. 1:**
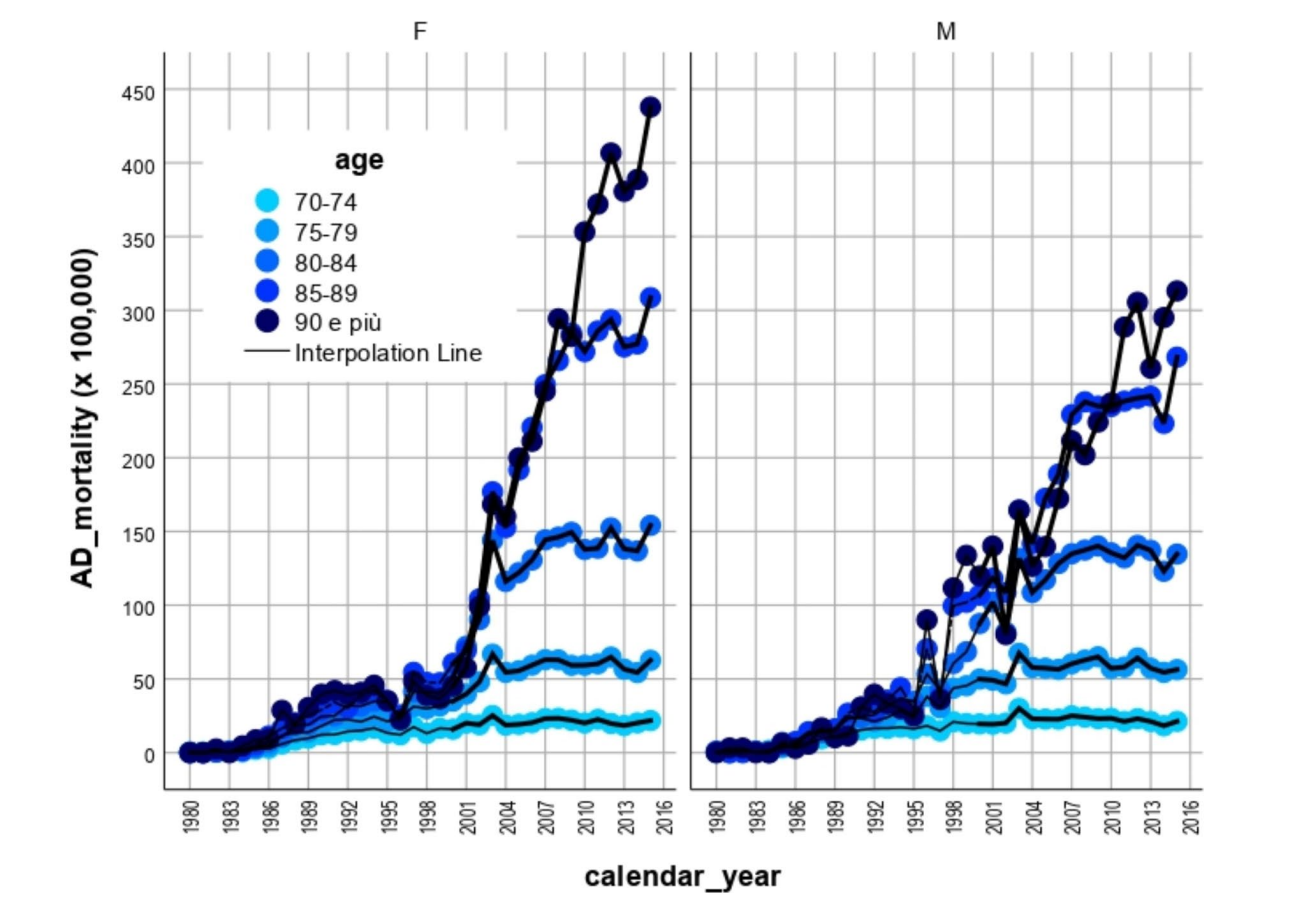
*Mortality rates (x 100*,*000) in the whole country*,* for males (right panel) and females (left panel) and by age class*,* multiplied by 100*,*000*

From 1980 to 2015, 153,213 deaths for AD were recorded (males 55,273, females 97,940) with a CR of 5.48 cases per 100,000 in males and 9.16 in females. Considering the AMR, females showed higher mortality during the whole study period, with 9.08/100,000 (CI 95%: 9.02–9.13) versus 8.86/100,000 in males (CI 95%: 8.78–8.93). Figure 1 shows mortality rates in the whole country along the study period, by classes of age (above 70 years) and sex. While rates were similar for males and females up to 80 years, mortality in females is greater above 80 years.

The analysis for geographical areas showed significantly lower SMRs than for whole country in North-East Italy for both sexes, whereas significantly higher SMRs were observed in Central Italy for both sexes, in North-West for males only and in the Islands for females only (Table 1).

**Table 1:** Number of deaths, crude rates (CR) per 100,000 persons/year, and standardized mortality ratios (SMR) with relative 95% confidence interval (95% CI) for the period 1980–2015, by sex and main geographical areas

	Area	*N*. deaths	CR x 100,000	SMR	95% CI
**Males**	*North-West*	15,102	5.68	103.23**	101.59–105.90
	*North-East*	9797	5.22	88.94*	87.18–91.73
	*Center*	12,503	6.46	105.55**	103.71-108.43
	*South*	11,632	4.75	99.73	97.93-102.57
	*Islands*	6239	5.37	101.97	99.45-105.54
	***ITALY***	55,273	5.48	***100***	***-***
**Females**	*North-West*	28,881	10.17	100.89	99.73-103.07
	*North-East*	17,062	8.57	81.59*	80.37–83.83
	*Center*	22,664	10.93	110.98**	109.54-113.44
	*South*	18,695	7.29	99.85	98.42–103.30
	*Islands*	10,638	8.70	114.93**	112.75-118.14
	***ITALY***	97,940	9.16	***100***	***-***

* SMR significantly lower than SMR for Italy

** SMR significantly higher than SMR for Italy

## Mortality trend analysis

**Fig. 2:**
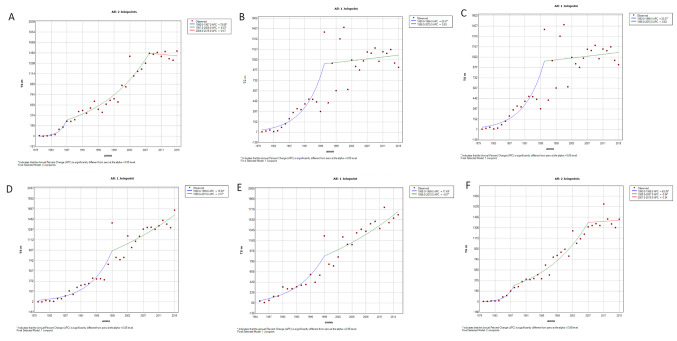
Mortality trend by geographic area estimated through the joinpoint regression analysis, in males. **A**: North-West Italy, **B**: North-East, **C**: Central Italy, **D**: South Italy, **E**: Islands, **F**: Italy

**Fig. 3:**
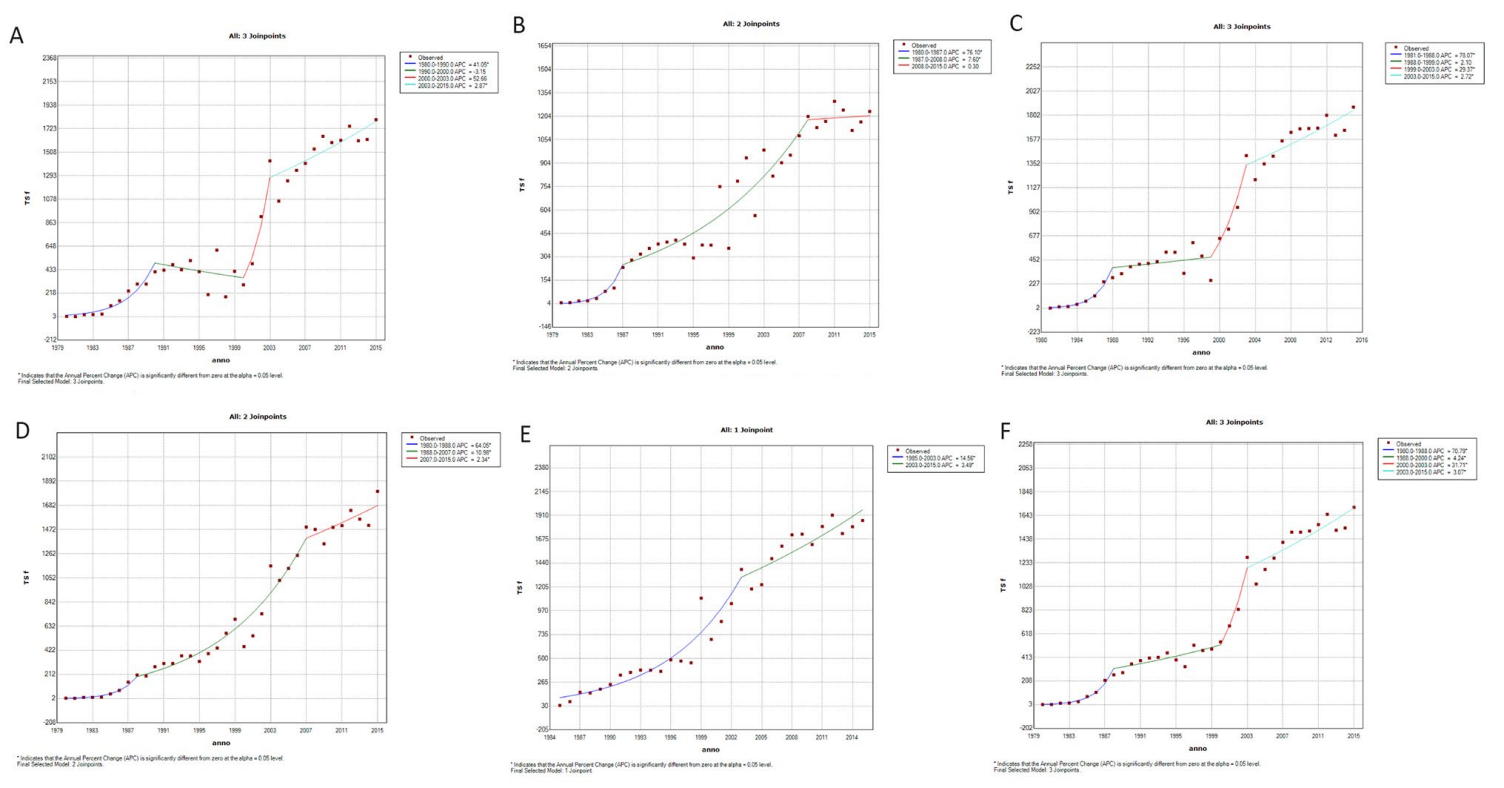
Mortality trend by geographic area estimated through the joinpoint regression analysis, in females. **A**: North-West Italy, **B**: North-East, **C**: Central Italy, **D**: South Italy, **E**: Islands, **F**: Italy

The linear regression analysis for whole Italy showed an increasing trend over all analysed period (males APC 17%, females APC 18.6%, both *p* < 0.05) (see Figs. 2 and 3, both showing annual AMR and modelled by regression analysis). In detail, an initial sharp increase up to 1988 was described for both sexes (males APC 70.8%, females APC 63.4%, both *p* < 0.05), followed by a slight increase for both sexes (up to 2007 for males APC 8.5%, up to 2000 for females APC 4.2%, both *p* < 0.05). Then, for males from 2007 to 2015 a plateau was observed (APC 0.3%, not significant); whereas for females another sharp upward trend was registered from 2000 to 2003 (APC 31.7%, *p* < 0.05) followed by a light increase (APC 3.1, *p* < 0.05).

The estimation of the mortality through the regression analysis showed the same increasing trend across the five geographical areas.

For males (Fig. 2), we observed an upward trend (APC in NW 17.6%, in NE 9.9%, in C 16.1%, in S 11.9%, in I 10.1%, all *p* < 0.05) with two patterns of mortality trend. The first pattern, observed in North-West and in Central Italy, reflects the same observed for the whole country with an initial sharp increase (until 1987 in NW APC 79.1%, until 1988 in C 63.7%, both *p* < 0.05), followed by a slight increase (until 2008 in NW APC 8.0, until 2007 in C APC 8.8%, both *p* < 0.05), and then a plateau (APC in NW -0.5%, in C 0.5%, both not significant). The second pattern, observed for North-East, South and the Islands, showed an initial sharp increase (until 1996 in NE APC 22.1%, until 1999 in S 19.5%, and in I 17.5%; all *p* < 0.05) followed by a plateau in North-East (APC 0.5%, not significant) or a modest increase in South and the Islands (S: APC 3.4%, I: APC 4.1%; both *p* < 0.05) (Fig. 2).

Regarding females (Fig. 3), the same increasing trend was observed in all geographical areas (APC in NW 14.5%, in NE 17.1%, in C 18.0%, in S 19.1%, in I 10.0%, all *p* < 0.05). We observed in almost all areas a sharp initial increase of mortality until around the year 1990 (41.1% in NW, 76.1% in NE, 78.1% in C, 64.1% in S, all *p* < 0.05) followed by a slight increase until the end of the analysed period (Fig. 3).

## Discussion

In our study, we observed a rising trend in AD mortality rates in Italy over the study period, which spans more than three decades (1980–2015). More specifically, we reported a consistent overall upward trend in mortality rates for both sexes. When considering mortality trends over time, a sharp increase was reported for both sexes up to 1988, followed by a period of more moderate growth until 2007 for males and until 2000 for females, specifically. Subsequently, males reached a plateau from 2007 to 2015, while females experienced another sharp increase from 2000 to 2003 followed by a slight rise (Figs. 2 and 3). The first increasing trend up to the end of the eighties was observed also for Parkinson’s disease in a previous study [8]. Our findings align with the rising trend observed in Europe for the period 1994–2013 (mortality rates increase of 4.7% and 6% in men and women, respectively), with some differences between eastern and northern countries (increase) and western countries (decrease) [9].

When examining mortality trends by sex and geographical regions, an upward trend was observed across all regions for both males and females. However, the increase in male mortality mirrored the national trend in North-West and Central Italy, but not in North-East, South, and the Islands, where it did not surge until the mid to late 1990s and it was then followed by either a plateau or a mild increase (Figs. 2 and 3). Future investigations should address in detail the possible role of regional confounding factors, as access to health services and their availability, socio-economic status or different socio-health policies, that may vary from one region to another.

The Italian mortality rising trend reflects the worldwide progressive increase of prevalence and incidence of neurodegenerative disorders, including AD [10, 11]. This increasing burden may be attributed to factors such as increasing longevity, better lifestyles, declining smoking rates, and growing industrialization [10, 12], and underscore the urgency of addressing the growing impact of AD on public health. However, higher mortality rates attributed to AD may not solely reflect an increase of AD cases but may also be indicative of other factors.

First, improvements in diagnostic accuracy. Advancements in neuroimaging techniques, biomarker assays, and clinical diagnostic criteria have enhanced our understanding of AD pathology, enabling earlier and more accurate diagnosis. As a result, individuals previously undiagnosed or misdiagnosed with other cognitive disorders may now be properly identified as probable AD cases, leading to a higher reported mortality rate attributable to the disease [13, 14].

Second, as diagnostic tools and criteria evolve, healthcare professionals become better equipped to identify probable AD as a contributing factor to mortality. This improved diagnostic precision allows for a more accurate assessment of AD-related mortality over time [15]. Therefore, the intersection of higher mortality rates and improved diagnostic accuracy underscores the importance of interpreting temporal trends in AD mortality within a broader context. While an apparent increase in mortality rates may raise concerns about the escalating burden of AD, it may also reflect positive strides in disease detection and diagnostic precision.

Third, environmental factors, such as demographic changes and seasonal variations, can also influence mortality rates. For instance, the highest mortality rate in some geographical areas in 2015 may be attributed to a combination of demographic phenomena, a particularly hot summer, and a severe flu season, that caused an increase of general mortality in Italy [16].

Fourth, issues related to death certificates represent a challenge in mortality studies. While death certificates contribute to proper coding and produce comprehensive mortality statistics, correctness of certification remains a difficult to eliminate limitation. Inappropriate death certification compilation may explain different mortality rates observed over time and geographical areas.

Fifth, changes in disease coding and registration criteria, such as switching from ICD-9 to ICD-10, may also have affected mortality trends, as already observed for Parkinson’s disease [8]. Despite these challenges, studies on temporal trends of mortality remain reliable, providing valuable insights into disease burden over time [17].

Finally, our study revealed a noteworthy finding regarding the lack of change in mortality due to AD despite the introduction of several drugs approved for its treatment in the late 1990s and early 2000s. These drugs include rivastigmine [18], donepezil [19], and memantine [20], that are based on distinct mechanisms of action and intended effects on cognitive symptoms associated with AD. Rivastigmine and donepezil are cholinesterase inhibitors, which work by increasing the levels of acetylcholine in the brain, a neurotransmitter involved in memory and learning processes [21, 22]. Memantine, on the other hand, is an N-methyl-D-aspartate (NMDA) receptor antagonist that regulates glutamate activity, aiming to reduce neuronal damage and improve cognitive function [23]. While the available evidence suggests that these symptomatic medications may improve cognitive function and quality of life in AD patients [24, 25], their impact on mortality rates remains unclear. However, our findings suggest that mortality trends due to AD in Italy during the study period were not influenced by the introduction of any of the aforementioned drugs. Indeed, they did not seem to play a relevant role in determining positive or negative changes in AD mortality trends, as instead observed for Parkinson’s disease after the introduction of dopamine agonists on the Italian market [8]. Unlike medications for Parkinson’s disease, that have shown to impact mortality [8, 26], the therapeutic landscape for AD primarily revolves around cognitive symptoms management. This inherent limitation in addressing the underlying pathology of AD may contribute to the observed lack of change in mortality rates. Future research will address whether anti-amyloid drugs aiming to target the underlying pathology of AD [27] will have an impact AD mortality trends.

Neuropsychiatric symptoms (irritability, agitation, sleep disturbances, delusions, hallucinations, aggression and impulsivity) commonly accompany dementia [28] and are often treated with antipsychotics, that have been linked with an overall increase of all-cause [29] and stroke-specific mortality [28]. Use of antipsychotic, however, may also increase the, and to propose innovative treatments that may help to slow down the disease progression risk for other adverse events as parkinsonian symptoms, thromboembolism or pneumonia [30]. In central Italy, the use of atypical antipsychotics in dementia has been found to increase the risk of mortality versus patients not taking them [31]. However, it remains difficult to disentangle whether the increase of mortality is due to a direct effect of the antipsychotic drugs, to their adverse effects, to the fact that patients requiring this therapy have generally more severe clinical pictures or to a combination of all these factors.

In conclusion, our study contributes to our understanding of AD mortality trends in Italy, providing valuable insights into the evolving landscape of the disease over time. As we work towards reducing the burden of AD and improving outcomes for affected individuals, it is crucial to acknowledge the complex nature of disease diagnosis and mortality assessment, and to propose innovative treatments that may help to slow down the disease progression [32].
